# Effect of pH on small-molecule inhibitor binding to influenza virus hemagglutinin

**DOI:** 10.1016/j.jbc.2026.111150

**Published:** 2026-01-10

**Authors:** Varada Anirudhan, Irina Gaisina, Amir Shimon, Hyun Lee, Saad Alqarni, Balaji Manicassamy, Terry W. Moore, Kai Xu, Michael Caffrey, Lijun Rong

**Affiliations:** 1 Department of Microbiology and Immunology, University of Illinois Chicago, Chicago, Illinois, USA; 2 Department of Pharmaceutical Sciences and UICentre, University of Illinois Chicago, Chicago, Illinois, USA; 3 Chicago BioSolutions Inc., Chicago, Illinois, USA; 4 Department of Biochemistry and Molecular Genetics, College of Medicine, University of Illinois Chicago, Chicago, Illinois, USA; 5 Biophysics Core at Research Resource Center, University of Illinois Chicago, Chicago, Illinois, USA; 6 Department of Pharmaceutical Chemistry, College of Pharmacy, University of Ha’il, Hail, Saudi Arabia; 7 Department of Microbiology and Immunology, University of Iowa, Iowa City, Iowa, United States; 8 UI Cancer Center, University of Illinois Chicago, Chicago, Illinois, USA; 9 Department of Veterinary Biosciences, The Ohio State University, Columbus, Ohio, USA; 10 Department of Microbial Infection and Immunity, The Ohio State University, Columbus, Ohio, USA

**Keywords:** antivirals, fusion inhibitors, hemagglutinin, influenza virus, mechanism of action, surface plasmon resonance, thermal shift assay

## Abstract

Influenza A viruses (IAVs) impose a tremendous socioeconomic burden, and the mainstay preventative strategy of using vaccines faces challenges related to annual reformulation and variable efficacy (30–70%). The occurrence of antiviral resistance to the current Food and Drug Administration–approved anti-influenza drugs further highlights the urgent need for novel therapeutics. Our research group previously identified and optimized potent small-molecule inhibitors targeting IAV’s hemagglutinin (HA), a surface glycoprotein crucial for viral entry and membrane fusion. Fusion occurs after the virus is taken up by endocytosis in the late endosomes under acidic conditions (pH ∼4.9–5.5). In this study, we report the biophysical characterization of two small-molecule inhibitors that bind to recombinant H3 and H7 HA proteins (phylogenetic group 2). These two compounds exhibited binding affinities (*K*_*D*_) ranging from ∼0.4 to 18.6 μM and significantly stabilized H7 HA based on thermal shift assay. Remarkably, lowering the pH from 7.2 to 6.2 resulted in up to a ∼267-fold increase in binding strength. Detailed analysis of the compound binding site suggested a potential role of the E97 side chain in enhancing affinity at lower pH. On the other hand, remodeling of the compound binding site because of propagated structural changes appears to be the most likely explanation. Collectively, these findings elucidate a pH-dependent mechanism of action for HA-targeting antivirals and underscore the importance of evaluating protein–ligand interactions under physiologically relevant conditions. This consideration is particularly important for viral proteins such as IAV HA that undergo pH-triggered conformational changes during the endosome-dependent viral entry.

Influenza virus infections can lead to severe pneumonia, and sometimes multiorgan failure (1) with seasonal epidemics causing tremendous socioeconomic loss each year (2, 3). These viruses, belonging to the family of Orthomyxoviridae, are classified into genera A, B, C, and D, with influenza A viruses (IAVs) having the greatest pandemic potential; this is largely because of their ability to genetically diversify themselves by antigenic drift and antigenic shift processes. IAVs have thus far caused four pandemics in the past ∼100 years, resulting in considerable socioeconomic burden (4). These include the 1918 “Spanish Influenza” (H1N1), “Asian Influenza” (H2N2) in 1957, “Hong Kong Influenza” (H3N2) in 1968, and the “Swine flu” pandemic (H1N1) in 2009 (5). The current circulating IAV strains that cause seasonal epidemics are less pathogenic versions of the pandemic H1N1 and H3N2 strains (6). The Centers for Disease Control and Prevention estimates that the 2025 flu epidemic season in the United States resulted in at least 24 million illnesses, 310,000 hospitalizations, and 13,000 deaths caused by influenza viruses.

There are two general strategies against IAV infections: vaccination and antiviral therapies. An existing, cost-effective strategy to protect the human population from the debilitating effects of influenza infections is vaccination (7). However, vaccine effectiveness can vary from 30% to 70%, occasionally even lower, and largely relies on patient age, their immune status, and how closely matched the circulating and vaccine strains are in that particular season (8). On the other hand, there are several antiviral therapies currently in clinical use. The adamantanes, amantadine and rimantadine, are M2 ion channel inhibitors that were approved by the Food and Drug Administration in 1976 and 1994, respectively, to treat influenza infections; however, these are no longer recommended by the Centers for Disease Control and Prevention because of the high prevalence of viral resistance against them (9). Neuraminidase inhibitors are another class of US Food and Drug Administration–approved anti-influenza drugs. They function by blocking the release of newly formed virions from infected cells (10, 11). Baloxavir marboxil (Xofluza) is a polymerase inhibitor of IAVs and makes up the third class of approved anti-influenza small-molecule drugs (12). In addition, Favipiravir (T-705), an RNA-dependent RNA polymerase inhibitor, is currently in phase III clinical trials as an antiflu drug (13). In the case of all the approved anti-influenza drugs, drug resistance has been reported (14, 15, 16). Thus, effective antiviral therapeutics with novel mechanisms of action that can be used as a single agent or in combination with others are urgently needed to treat influenza infections.

The first step in the replication cycle of IAVs is the low-affinity multivalent binding of hemagglutinin (HA), a homotrimeric glycoprotein found on the IAV viral envelope, to sialic acid–containing glycans on the host cell (17). This attachment triggers uptake of IAVs by endocytosis, wherein the acidification within endosomes leads to fusion between the host membrane and the virus particle at a low pH; the value of this pH ranges between ∼4.9 and 5.5, and it depends on the virus strain as well as the internal and external host cell environments (18). From the point of entry until successful fusion, HAs undergo a series of reversible conformational changes, until finally, at the pH of trigger, an irreversible structural rearrangement occurs (Fig. 1*A*). Fusion results when the two separate monomers of HA, HA1 and HA2, partially dissociate from each other, followed by the insertion of a hydrophobic N-terminal fusion peptide into the endosomal membranes, which leads to fusion of the viral and endosomal membranes. Consequently, viral ribonucleoproteins are released into the cytoplasm, after which viral replication follows upon transport into the nucleus.

**Figure 1:**
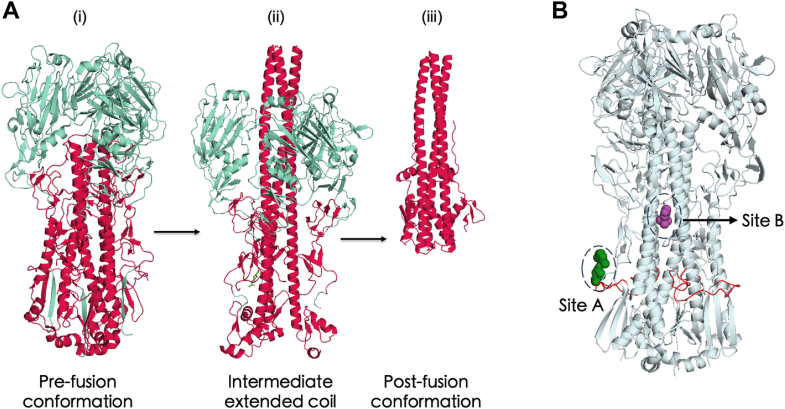
**Conformational changes of IAV hemagglutinin (HA) and binding regions of HA-targeting small molecule inhibitors.***A*, structure of HA (HA1 domain shown in *red* and HA2 domain shown in *teal*) (i) at pH 7.2, prefusion conformation (ii) at pH ∼5.2, known as extended coil (iii) at fusion pH (∼4.9–5.5), the postfusion conformation. *B*, structure of HA (*gray*) showing the known binding sites of the group-specific fusion inhibitors. Shown in *green* is CBS1117, a group 1 inhibitor (PDB entry: 6VMZ) and in *purple* is TBHQ (PDB entry: 3YEK), a group 2 inhibitor; the fusion peptide consisting of 20 amino acids is shown in *red*. Sites A and B correspond to the binding regions for group 1- and group 2-specific HA inhibitors, respectively. IAV, influenza A virus; PDB, Protein Data Bank; TBHQ, tertiary butylhydroquinone.

IAVs are classified into two subtypes based on the antigenicity of their HA glycoproteins. There are 18 HA subtypes consisting of group 1 (H1, H2, H5, H6, H8, H9, H11, H12, H13, and H16) and group 2 (H3, H4, H7, H10, H14, and H15) (19). In addition, H19, recently identified in common pochards in Kazakhstan, has not yet been antigenically classified (20). Small-molecule inhibitors directly targeting the HA proteins have been reported. Several small-molecule inhibitors that block the viral fusion process by hindering HA conformational change at low pH have been developed. Most, if not all, of these “fusion inhibitors” demonstrate group-selective inhibitory activities. For example, F0045(S), JNJ4796, and CBS1117 strongly inhibit group 1 HA activity and are all thought to bind to a common region in HA, but they do not inhibit group 2 HA (21, 22, 23). On the other hand, tertiary butylhydroquinone and its derivatives and *N*-cyclohexyltaurine show activity against group 2 HA; tertiary butylhydroquinone and *N*-cyclohexyltaurine, through cocrystal structural studies with group 2 HA, have been shown to bind to the same hydrophobic pocket in its stalk region, which is predominantly involved in viral fusion (Fig. 1*B*) (24, 25, 26, 27). Notably, group 1 and group 2 inhibitors appear to bind to different regions of the HA stalk, as depicted by sites A and B in Figure 1*B*. Consequently, a deeper understanding of drug–protein interactions at the molecular level is crucial in overcoming this existing barrier of subtype specificity.

We previously reported two group 2 HA small-molecule inhibitors that have an imidazopyrimidine scaffold with pseudovirus EC_50_ values in the micromolar range (28, 29, 30). To better understand the mechanism of action of these inhibitors and to provide guidance for further structure-based drug design, we have characterized the binding properties of two group 2-specific inhibitors amongst this series (Table 1; Fig. 2*A*) by thermal shift assays (TSAs) and surface plasmon resonance (SPR) in the present work.

**Table 1:** Antiviral characteristics of the influenza HA group–specific inhibitors (28))

Name	H5 EC_50_ (μM)^a^	H7 EC_50_ (μM)^b^	Pseudovirus (SB value)^c^
ING-1636	34.10 ± 1.21	0.31 ± 0.03	193.19^⊤^
SA-67	33.11 ± 3.70	0.04 ± 0.01	1347^⊤^

a EC_50_ values were obtained from pseudotyped H5 infection assay in A4549 cells.

b EC_50_ values were obtained from pseudotyped H7 infection assay in A549 cells.

c Selectivity bias (SB) values = 50% cytotoxicity in A549 cells/IC_50_ against ^⊤^H7 pseudotyped virus.

**Figure 2:**
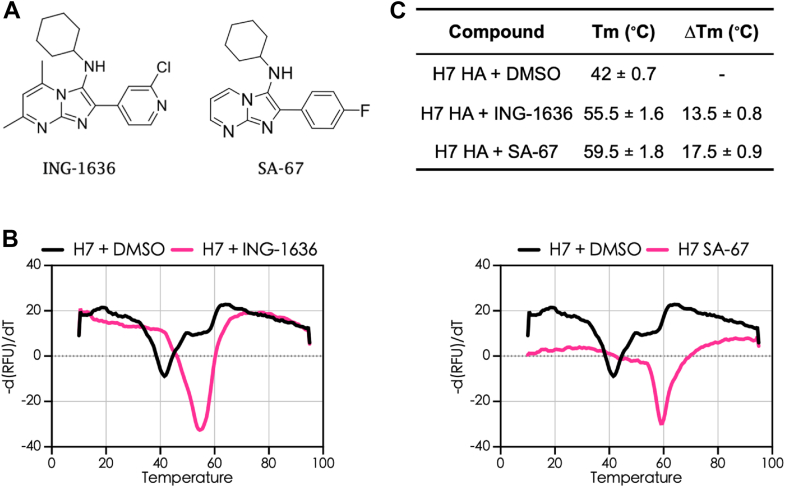
**IAV HA group 2-targeting small-molecule inhibitors.***A*, chemical structures of the IAV HA group 2–specific inhibitors. *B*, thermal shift assay with H7 HA recombinant proteins. *C*, ΔT_m_ values from thermal shift assay using recombinant H7 HA. HA, hemagglutinin; IAV, influenza A virus.

## Results

### Thermal shift analysis demonstrates stabilization of H7 HA by small-molecule inhibitors

Recombinant H3 and H7 (group 2 HA) proteins were expressed using an insect cell system as described previously (31). In each case, the HA constructs contained a C-terminal foldon domain to stabilize the trimeric state, followed by a polyhistidine sequence for purification and immobilization (31). Our mechanism of action studies started with TSA to qualitatively check for the binding of these group-specific small molecules to the HA protein. TSA uses an RT–PCR instrument for the measurement of change in fluorescence intensity, correlated to the melting temperature (*T*_*m*_) of a protein, in the absence and presence of a small-molecule binder. An indication of the compound binding to the protein and its stabilization is an increase in protein *T*_*m*_, whereas a decrease in *T*_*m*_ indicates destabilization of the protein. We observed that the group 2–specific small molecules ING-1636 and SA-67 increased the *T*_*m*_ of H7 HA by 13 and 17.5 °C, respectively, and that the change in *T*_*m*_ was positively correlated with their antiviral potencies (Fig. 2, *B* and *C*). Therefore, using TSA, we provide evidence that these compounds bind to and stabilize the HA protein.

### SPR reveals distinct binding kinetics and affinities of HA inhibitors toward group 2 HA

**Figure 3:**
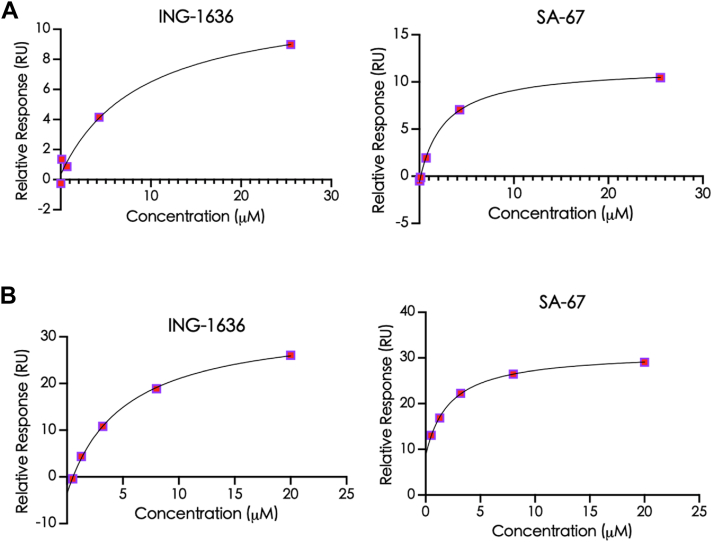
Steady-state binding of HA-targeting compounds with (*A*) H3 HA and (*B*) H7 HA from SPR experiments. HA, hemagglutinin; SPR, surface plasmon resonance.

SPR was employed to quantitatively study the binding interactions between the HA inhibitors and their respective binding partners, H3 and H7 HA. SPR entails performing a concentration-dependent binding experiment wherein the protein is immobilized on a gold sensor chip and small molecules are passed over it in real time; the goal of an SPR run is to determine the kinetic parameters, association (*k*_a_ or *k*_on_) and dissociation rate constants (*k*_*d*_ or *k*_off_), and the binding affinity (*K*_*D*_) (32). The *k*_on_ of the two compounds, ING-1636 and SA-67, binding to H3 HA was 2.51 × 10^5^ and 5.81 × 10^5^ M^-1^s^-1^, respectively, and *k*_off_ was very fast for both compounds (Fig. S1 and Table 2). On the other hand, the *k*_off_ of ING-1636 and SA-67 binding to H7 HA exhibited much slower dissociation rates (Fig. S1 and Table 2). Specifically, *k*_off_ rates change from an average of 4.08 s^-1^ with H3 HA to 3.68 × 10^-2^ s^-1^ with H7 HA. Binding affinity (*K*_*D*_) values of these compounds, which range from 0.4 to 18.6 μM, indicated high-affinity interactions in the low micromolar and nanomolar ranges (Fig. 3; Table 2). Specifically, ING-1636 and SA-67 bind to H7 HA with stronger affinities (2.4-fold and 10-fold, respectively). The drug-target residence time, which is calculated as the reciprocal of *k*_off,_ represents the drug’s lifetime for target binding and is an important parameter that is now increasingly considered as an indication of a drug with favorable *in vivo* pharmacodynamic (PD) properties (33). The residence times for our small molecules were 19.42 and 53.82 s for H7 HA, suggesting they reside in the binding pocket with favorable residence times (Table 2). Their binding parameters to H3 HA indicated poorer kinetic favorabilities, with residence times (0.21 and 0.40 s) (Table 2). Overall, our SPR studies demonstrate relatively strong binding affinities for these small molecules interacting with HA, with significant differences in the binding kinetic parameters, which is intriguing because they are all based on the same scaffold.

**Table 2:** Rate constants and binding affinity values of group 2–specific compounds binding to H3 HA and H7 HA

Compound	*k*_on_ (1/Ms)	*k*_off_ (1/s)	Residence time (s)	*K*_*D*_ (μM)
ING-1636				
H3 HA	2.51 (±0.64) × 10^5^	5.43 (±2.2)	0.21 ± 0.14	18.61 ± 9.62
H7 HA	6.62 (±0.37) × 10^3^	5.21 (±0.04) × 10^-2^	19.42 ± 0.13	7.82 ± 0.74
SA-67				
H3 HA	5.81 (±0.67) × 10^5^	2.73 (±0.2)	0.40 ± 0.00	4.00 ± 2.00
H7 HA	2.83 (±1.41) × 10^4^	2.14 (±0.07) × 10^-2^	53.82 ± 17.1	0.41 ± 0.03

### pH-Dependent modulation of HA inhibitor binding explains discrepancies between SPR-derived *K*_*D*_ and pseudovirus EC_50_ values

**Figure 4:**
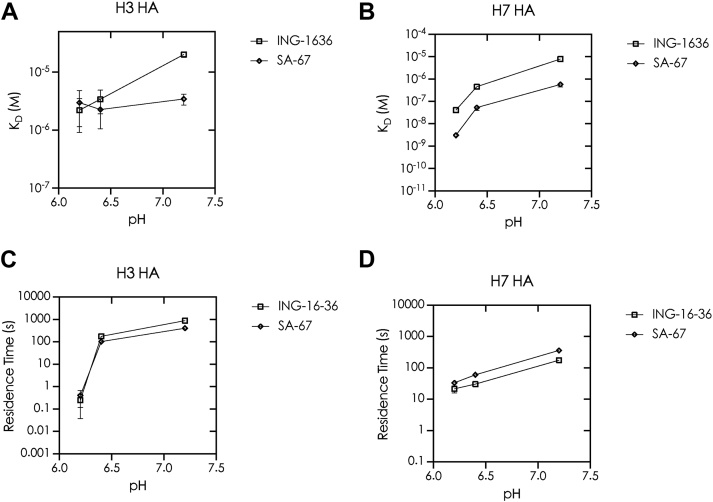
**The pH dependence of the binding (*K*_*D*_) of small molecules ING-1636 and SA-67 as shown by SPR using recombinant.** (*A*) H3 HA and (*B*) H7 HA protein. An increase in residence time values was observed upon lowering the pH for ING-1636 and SA-67 with (*C*) H3 HA and (*D*) H7 HA. HA, hemagglutinin; SPR, surface plasmon resonance.

From our SPR experiments, we noted significant discrepancies between the *K*_*D*_ values and pseudovirus EC_50_ values for the compounds ING-1636 and SA-67, with ratios of *K*_*D*_/EC_50_ ranging from 10 to 25 for H7 HA (Table 3). As mentioned earlier, the endosomal acidification, HA, is known to undergo a series of reversible conformational changes before an irreversible modification occurs at low pH, resulting in membrane fusion (34, 35, 36). We thus postulated that pH effects within the endosome could affect the *K*_*D*_ in some cases. For example, significantly enhanced binding within the endosome (*i.e*., reduced *K*_*D*_) could result in a lower apparent EC_50_ and, conversely, significantly impaired binding (*i.e*., increased *K*_*D*_) within the endosome could result in a higher apparent EC_50_. Since the compounds are also exposed to the acidic environment of late endosomes, we aimed to better mimic physiological conditions in our biophysical assays and assess whether binding affinities measured at lower pH more closely correspond with pseudovirus EC_50_ values. Accordingly, we tested this notion by running SPR experiments with buffers at pH 7.2, 6.4, and 6.2 for the two compounds, where there were very significant discrepancies between the EC_50_ and *K*_*D*_ values. From our analyses, we observed that, in the case of H3 HA, the *K*_*D*_ values reduced 14.3-fold from 18.6 to 1.3 μM for ING-1636 and that of SA-67 reduced ∼2.9-fold from 4.0 to 1.4 μM when pH was reduced from 6.2 to 7.2 (Fig. 4*A*; Table 4; Figs. S2 and S3). From our SPR experiments performed at different pH levels with H7 HA, the *K*_*D*_ values reduced 42-fold from 2.1 to 0.05 μM for ING-1636 and that of SA-67 reduced 266.7-fold from 0.8 to 0.003 μM (Fig. 4*B*; Table 4; Figs. S2 and S3). Lowering the pH, in all cases, significantly increased the residence time as well (Figs. 4, *C* and *D*, and S3; Table 4). We observed a ∼4400- and ∼1016-fold increase in the residence time of ING-1636 and SA-67 binding to H3 HA, respectively, and a 9- and 11-fold increase in the residence time of ING-1636 and SA-67 binding to H7 HA, respectively. Overall, it is evident that the lower pH of the endosome could enhance binding of these compounds to HA, which, in the case of ING-1636 and SA-67, resulted in better correspondence with the pseudovirus EC_50_.

**Table 3:** (*K*_*D*_/EC_50_) of the compounds at pH 7.2

Compound	*K*_*D*_	EC_50_^a^	*K*_*D*_/EC_50_^a^
ING-1636	7.82 ± 0.74	0.31 ± 0.03	25.22
SA-67	0.41 ± 0.03	0.04 ± 0.01	10.25

a K_*D*_ from SPR with H7 HA; EC_50_ from H7 pseudovirus assay.

**Table 4:** *K*_*D*_ values (M) and residence time (s) from SPR runs with H3 HA or H7 HA at pH values 7.2, 6.4, and 6.2

Recombinant protein & pH values of the SPR buffer	*K*_*D*_ (μM)	Fold improvement	Residence time (s)	*K*_*D*_ (μM)	Fold improvement	Residence time (s)
	ING-1636			SA-67		
H3 HA						
pH 7.2	18.6 ± 1.0	NA	0.2 ± 0.1	4.0 ± 0.5	NA	0.4 ± 0.0
pH 6.4	4.5 ± 0.6	4.2	176.4 ± 11.6	1.7 ± 0.3	2.4	102.0 ± 7.2
pH 6.2	1.3 ± 0.2	14.4	880.7 ± 58.6	1.4 ± 0.3	2.8	406.5 ± 32.6
H7 HA						
pH 7.2	2.1 ± 0.3	NA	19.6 ± 0.1	0.8 ± 0.03	NA	33.0 ± 7.1
pH 6.4	0.6 ± 0.08	4.2	29.9 ± 2.5	0.04 ± 0.007	19.4	61.5 ± 5.5
pH 6.2	0.05 ± 0.007	46.8	177.3 ± 13.6	0.003 ± 0.000	306.5	363.0 ± 33.1

NA, not applicable.

Also shown are the fold difference between the *K*_*D*_ values and H7 pseudovirus EC_50_ (*K*_*D*_/IC_50_) (“fold improvement”) with decreasing pH.

### Structure-based understanding of pH dependence of the binding of compounds ING-16-36 and SA-67

**Figure 5:**
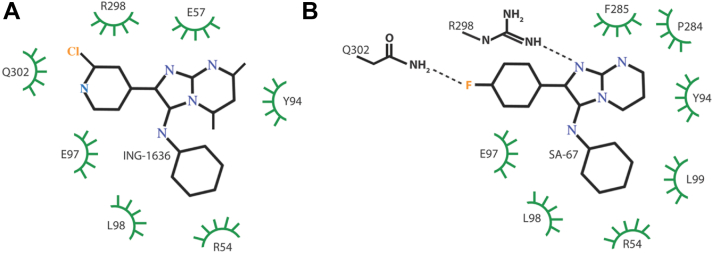
Interactions of inhibitors based on Ligplot analysis of recent cryo-EM structures of influenza H7 HA in complex with ING-1636 (*A*) and SA-67 (*B*). Hydrogen bonds are depicted as *dotted lines*, and hydrophobic interactions are depicted as *green circles*. Analysis is based on the structures by Xu *et al.*, 2025 (PDB entries: 9OO1 and 9ONZ). HA, hemagglutinin; PDB, Protein Data Bank.

Based on the structures of the small molecules ING-1636 and SA-67 (Fig. 2*A*), there are no ionizable groups in the pH range tested (the predicted p*K*a for the cyclohexyl amine are 3.6 and 5.4 for ING-1636 and SA-67, respectively), and thus, the pH effects observed for the two compounds most likely stem from changes in the ionization state of the protein. For example, the favorable binding at lower pH, representative of the endosome, could stem from direct interactions or indirect structural effects on the binding pocket. Recently, our groups have determined the cryo-EM structures of these compounds individually in complex with influenza H7 HA at 2.8 Å and pH 7.4 (37), and thus, a structure-based analysis is merited. It is well established that histidine protonation causes conformational changes in HA with reversible changes at intermediate pH, followed by an irreversible change at ∼pH 5.5 (38). However, there are no histidine residues actively involved in the binding site or in close proximity (the closest histidine, H450, is >12 Å from the compounds). Using the program LigPlot (39), which identifies interactions between a ligand and its target protein, we analyzed the amino acid interactions of ING-1636 and SA-67 with HA. As shown in Figure 5*A*, ING-1636 exhibits multiple hydrophobic interactions in the binding site. A similar set of hydrophobic interactions are also observed for SA-67 with the addition of two potential hydrogen bonding interactions between the Q302 side chain and the SA-67 fluorine and the R298 side chain and the N1 nitrogen of the imidazopyrimidine of SA-67 (Fig. 5*B*). Interestingly, E97 forms hydrophobic interactions with the compounds in both cases and presents a potential residue that could become protonated in the pH region of the experiments. For example, the average p*K*_a_ of glutamic acid side chain is 4.2 in proteins; however, the observed range is 2.1 to 8.8, with the higher values often occurring in hydrophobic environments (40). Thus, the more favorable binding properties of the two compounds at low pH could be due to enhanced hydrophobic interactions with the E97 side chain. Nonetheless, protonation of distant groups that cause remodeling of the compound binding site may also play important roles in the enhanced affinity.

## Discussion

Targeting IAV HA glycoprotein is an effective therapeutic strategy based on the criticality of this protein for membrane fusion and viral entry (41). Toward our goal of developing entry inhibitors against IAVs, we have synthesized several small-molecule inhibitors, which are highly potent against different IAVs. In this report, we characterized the binding properties of two entry inhibitors for group 2 IAV subtypes, one primarily circulating in humans and one primarily circulating in avians with potential zoonotic transfer to humans. Using TSA and SPR, we provide biophysical evidence for their strong binding to HA. Interestingly, HA binding of two of the inhibitors is strongly dependent on pH, with significant enhancement of binding affinity at pH values found in the late endosomal compartment. In addition, the initial high-throughput screen hit compound published earlier for compound CBS1194, which has the same imidazopyrimidine chemical scaffold as the tested compounds, prevented the trypsin-mediated cleavage of group 2 HA at low pH (30). Together with the structural insights of complexes of two small-molecule inhibitors with the same core scaffold (37), the data suggest that these small molecules act by stabilizing the prefusion HA conformation and thereby preventing successful viral fusion.

Using SPR with recombinant H3 and H7 HA, we conducted real-time kinetic analyses of binding of the two HA group 2–specific compounds, ING-1636 and SA-67. Our experiments indicated fast association rates of the tested compounds binding to H3 and H7 HA. This is encouraging since the on-rate, *k*_on_, has been reported to be associated with *in vivo* target occupancy and also target rebinding (42, 43); in addition, a fast *k*_on_ is positively correlated to the speed of drug action on the target as well (44).

While the equilibrium binding metric *K*_*D*_ and EC_50_ values from *in vitro* assays are important in evaluating a drug’s potential use in treating humans, in living organisms, several physiological factors influence its true binding affinity (45). These include tissue uptake and distribution, gastrointestinal absorption, hepatic and renal detoxification; these characteristics result in ligand concentration variation under physiological conditions, and thus, human bodies are considered to be “open systems” (46). In such systems, the concentration-independent off-rate (*k*_off_) is considered clinically relevant, and a crucial determinant of drug success is residence time, the inverse of *k*_off_ (33). From our studies, the residence time for group 2 compounds with H7 HA was favorable (∼20–54 s), indicating the likelihood of favorable drug characteristics in future *in vivo* PD studies (47). Our data also suggest that these drugs may have a better PD profile acting against H7 HA as compared with H3 HA (residence time = 0.21–0.40 s). Future work using IAV (H3 or H7 strains) challenges in animal models is needed to evaluate the physiological consequences of these drugs' inhibitory efficacies based on their *in vitro* residence times.

Within the first 30 min of IAV entry, the viral glycoprotein HA undergoes protonation of amino acid residues in the acidifying environment of early endosomes, when finally, at pH ∼4.9 to 5.5 (the exact value is HA strain–dependent and relates to pathogenicity (48)) in the late endosome, an irreversible structural reorganization of HA facilitates fusion between viral and host endosomal membrane (34). Once the HA-targeting drug is bound to its respective protein, it is also exposed to the low pH environment of the endosomes; thus, changes in the charges of its ionizable groups and structural perturbations occur. If a small molecule demonstrates significantly lower affinity to HA and shorter residence time at pH lower than 7.2, it may indicate a loss of inhibitory action as the virus goes through environmental pH alterations in the endosome. Our SPR studies with buffers at lower-than-neutral pH provide evidence for pH-dependent binding for the two compounds ING-1636 (H3 and H7 HA) and SA-67 (H3 and H7 HA). Our rationale behind exploring this avenue was our observation that their *K*_*D*_ and EC_50_ values were strikingly different from one another. Our data indicated that the pH of the binding conditions is a key determinant of the binding constant.

Differences between the equilibrium constants EC_50_ and *K*_*D*_ for the same ligand–protein complexes have, in fact, been previously reported in binding studies with HA and in the case of other antivirals as well—although the cause was not well understood. For instance, the *K*_*D*_ of MBX2546 with PR8 H1 as measured by TSA was 5.3 μM, 18-fold greater than its IC_50_ from infectious virus assays (0.3 μM) (49). White *et al.* (50) reported that a H1 HA–targeting compound, S20, showed a *K*_*D*_ value, as measured by biolayer interferometry, which was 35-fold higher than its IC_50_ (5.29 μM *versus* 0.15 μM). A *K*_*D*_ value 42-fold less than its IC_50_ was reported for another H1-binding compound, ginsenoside RK1 (51).

IAV HA is a structurally dynamic protein, which is metastable at neutral pH, that undergoes several subtle conformational changes during its course of entry into the cell (17, 52). HA exists in many reversible intermediate conformational states throughout the process of entry and fusion, and indeed, in our SPR experiments, we see that the binding characteristics at pH 7.2 were retained even after the protein was exposed to pH 6.2 for both H3 and H7 HA (34, 53, 54). Our data also signify the requirement for medicinal chemists to consider the IAV HA protein’s structural conformation at pH values lower than 7.2 while designing antiviral drugs, which, to our knowledge, is a novel perspective. For pH-dependent fusion proteins such as HA, Ebola glycoprotein GP2 (55), and severe acute respiratory syndrome coronavirus 2 spike protein (56), we believe this can be an important criterion for strategic drug design. Similar studies conducted with other small molecules and HA proteins of other group 1 and group 2 strains and with other fusion proteins can add more valuable information in this regard.

Drug discovery is a cumbersome task, with the degree of success being considerably low, and the scientific community now agrees, based on substantial evidence, that equilibrium binding metrics measured under conditions of a closed system are less reliable in predicting dynamic *in vivo* protein–drug interactions (33, 57, 58). The assessment of a target–drug interaction’s kinetic signature parameters, *k*_on_, *k*_off,_ residence time, and *K*_*D*_ can be helpful in reducing drug attrition rates and, toward that end, SPR is a powerful technique to study the binding events in detail, as we have shown in this study (59, 60).

## Experimental procedures

### Cell culture

293T embryonic kidney cells (American Type Culture Collection, #CRL-1573) and human A549 lung epithelial cells (American Type Culture Collection, #CCL185) were cultured in Dulbecco's modified Eagle's medium supplemented with 10% (v/v) fetal bovine serum (Gibco), 100 units of penicillin, and 100 μg/ml streptomycin (Invitrogen) at 37 °C and 5% CO_2_.

### Viral entry assays

As described previously, viral entry assays to evaluate EC_50_ and CC_50_ values of the small-molecule inhibitors were done using pseudoviruses containing H7 HA (A/Anhui/1/2013 [H7N9]) and neuraminidase N1 (NA), and the HIV-1 proviral vector containing luciferase gene (pNL4-3.Luc.R-E− from National Institutes of Health AIDS Research and Reference Reagent Program) (30). On 96-well plates, low-passage A549 cells were seeded at 5000 cells/well cell density and incubated at 37 °C and 5% CO_2_ for 24 h prior to pseudovirus infection. Drugs, resuspended in DMSO, were tested in the range of 100 μM to 5.1 nM. Post addition of pseudovirus with drug or DMSO (DMSO concentration did not exceed 1% [v/v]), viral infection was quantified based on luminescence using Neolite reporter system (Revvity). Data were normalized to virus with 1% DMSO. Drug cytotoxicity was evaluated using the CellTiter-Glo Luminescent Cell Viability Assay (Promega). The values of EC_50_ and CC_50_ were calculated by fitting dose–response curves with four-parameter logistic regression in GraphPad Prism (GraphPad Software, LLC) version 10.6.0.

### Thermal shift assay

Recombinant H3 and H7 HA proteins were prepared as described previously by expression in SF9 insect cells cotransfected with a pAcGP67 plasmid containing either H3 HA or H7 HA expression construct and BD BaculoGold linearized baculovirus DNA (BD Biosciences) (22, 36). All TSA or differential scanning fluorimetry experiments were performed using a Bio-Rad CFX Duet RT–PCR machine. Done in duplicates, each reaction had a total volume of 25 μl and included 5X SYPRO Orange dye (Sigma), 100 μM inhibitor (or DMSO only as a negative control), and 1 μM H7 HA protein dissolved in buffer (50 mM Pipes at pH 7.2 with 150 mM NaCl). The temperature was increased from 25 °C to 95 °C at the rate of 0.075 °C/s, and the fluorescence intensity was recorded in real time as relative fluorescence units. First derivatives for each measurement were calculated using the Bio-Rad CFX software. Melting temperature (*T*_m_) values were determined from dF/dT *versus* T plots.

### Surface plasmon resonance

All SPR experiments were conducted using the Biacore T200 (Cytiva) instrument with a Xantec NiHC200M-NTA Chip at 25 °C. Purified, His-tagged H3 or H7 HA protein at a concentration of 80 μg/ml was injected and immobilized on the chip (immobilization buffer was composed of 10 mM Hepes, 150 mM NaCl, and 0.005% v/v surfactant P20); 1-ethyl-3-(3-dimethylaminopropyl) carbodiimide–*N*-hydroxysuccinimide coupling after His-capture step for immobilization was done, and an immobilization level of at least 9000 relative unit was achieved. A ligand-free reference flow cell (FC1) was prepared by activation and ethanolamine blocking and used for in-line reference subtraction (reporting FC2–FC1). The binding buffer used was PBS with 0.05% Tween-20 at pH 7.2, 6.4, or 6.2. Injected analyte concentrations were 20, 3.3, 0.55, 0.06, and 0.006 μM. The data were fit using the steady-state affinity model (GraphPad Prism, version 10.6.0) to evaluate *K*_*D*_ values, and the Biacore Insight Evaluation software was used to fit the kinetic data (1:1 Langmuir model) to evaluate *k*_on_ and *k*_off_ values. All the kinetic parameters of drug–protein interactions were determined as average values of technical duplicates.

### Data availability

All data are included in this article and in the document containing supporting information.

## Supporting information

This article contains supporting information.

## Conflict of interest

The authors declare that they have no conflicts of interest with the contents of this article.

## References

[1] Sellers S.A., Hagan R.S., Hayden F.G., Fischer W.A. (2017). The hidden burden of influenza: a review of the extra-pulmonary complications of influenza infection. Influenza Other Respir. Viruses.

[2] Somes M.P., Turner R.M., Dwyer L.J., Newall A.T. (2018). Estimating the annual attack rate of seasonal influenza among unvaccinated individuals: a systematic review and meta-analysis. Vaccine.

[3] Putri W.C.W.S., Muscatello D.J., Stockwell M.S., Newall A.T. (2018). Economic burden of seasonal influenza in the United States. Vaccine.

[4] Charostad J., Rezaei Zadeh Rukerd M., Mahmoudvand S., Bashash D., Hashemi S.M.A., Nakhaie M. (2023). A comprehensive review of Highly Pathogenic Avian Influenza (HPAI) H5N1: an imminent threat at doorstep. Trav. Med. Infect. Dis.

[5] Short K.R., Richard M., Verhagen J.H., Van Riel D., Schrauwen E.J.A., Van Den Brand J.M.A. (2015). One health, multiple challenges: the inter-species transmission of influenza A virus. One Health.

[6] Taubenberger J.K., Kash J.C. (2010). Influenza virus evolution, host adaptation, and pandemic formation. Cell Host Microbe..

[7] Malosh R.E., McGovern I., Monto A.S. (2023). Influenza during the 2010–2020 decade in the United States: seasonal outbreaks and vaccine interventions. Clin. Infect. Dis..

[8] Wiggins K.B., Smith M.A., Schultz-Cherry S. (2021). The nature of immune responses to influenza vaccination in high-risk populations. Viruses.

[9] Deyde V.M., Xu X., Bright R.A., Shaw M., Smith C.B., Zhang Y. (2007). Surveillance of resistance to adamantanes among influenza A(H3N2) and A(H1N1) viruses isolated worldwide. J. Infect. Dis..

[10] Mckimmbreschkin J. (2000). Resistance of influenza viruses to neuraminidase inhibitors — a review. Antivir. Res..

[11] Moscona A. (2009). Global transmission of oseltamivir-resistant influenza. N. Engl. J. Med..

[12] Dufrasne F. (2021). Baloxavir marboxil: an original new drug against influenza. Pharmaceuticals.

[13] Shiraki K., Daikoku T. (2020). Favipiravir, an anti-influenza drug against life-threatening RNA virus infections. Pharmacol. Ther..

[14] Checkmahomed L., M’hamdi Z., Carbonneau J., Venable M.-C., Baz M., Abed Y. (2020). Impact of the Baloxavir-resistant polymerase acid I38T substitution on the fitness of contemporary influenza A(H1N1)Pdm09 and A(H3N2) strains. J. Infect. Dis..

[15] Takashita E., Kawakami C., Morita H., Ogawa R., Fujisaki S., Shirakura M. (2019). Detection of influenza A(H3N2) viruses exhibiting reduced susceptibility to the novel cap-dependent endonuclease inhibitor baloxavir in Japan, December 2018. Euro Surveill..

[16] Goldhill D.H., Te Velthuis A.J.W., Fletcher R.A., Langat P., Zambon M., Lackenby A. (2018). The mechanism of resistance to favipiravir in influenza. Proc. Natl. Acad. Sci..

[17] Skehel J.J., Wiley D.C. (2000). Receptor binding and membrane fusion in virus entry: the influenza hemagglutinin. Annu. Rev. Biochem..

[18] Daniels R. (1985). Fusion mutants of the influenza virus hemagglutinin glycoprotein. Cell.

[19] Compans R.W., Orenstein W.A., Vaccines for Pandemic Influenza (2009).

[20] Karakus U., Mena I., Kottur J., El Zahed S.S., Seoane R., Yildiz S. (2024). H19 influenza A virus exhibits species-specific MHC class II receptor usage. Cell Host Microbe..

[21] Yao Y., Kadam R.U., Lee C.-C.D., Woehl J.L., Wu N.C., Zhu X. (2020). An influenza A hemagglutinin small-molecule fusion inhibitor identified by a new high-throughput fluorescence polarization screen. Proc. Natl. Acad. Sci..

[22] Antanasijevic A., Durst M.A., Cheng H., Gaisina I.N., Perez J.T., Manicassamy B. (2020). Structure of Avian influenza hemagglutinin in complex with a small molecule entry inhibitor. Life Sci. Alliance.

[23] Van Dongen M.J.P., Kadam R.U., Juraszek J., Lawson E., Brandenburg B., Schmitz F. (2019). A small-molecule fusion inhibitor of influenza virus is orally active in mice. Science.

[24] Russell R.J., Kerry P.S., Stevens D.J., Steinhauer D.A., Martin S.R., Gamblin S.J. (2008). Structure of influenza hemagglutinin in complex with an inhibitor of membrane fusion. Proc. Natl. Acad. Sci..

[25] Antanasijevic A., Cheng H., Wardrop D.J., Rong L., Caffrey M. (2013). Inhibition of influenza H7 hemagglutinin-mediated entry. PLoS One.

[26] Antanasijevic A., Hafeman N.J., Tundup S., Kingsley C., Mishra R.K., Rong L. (2016). Stabilization and improvement of a promising influenza antiviral: making a PAIN PAINless. ACS Infect. Dis..

[27] Kadam R.U., Wilson I.A.A. (2018). Small-molecule fragment that emulates binding of receptor and broadly neutralizing antibodies to influenza A hemagglutinin. Proc. Natl. Acad. Sci..

[28] Alqarni S., Cooper L., Galvan Achi J., Bott R., Sali V.K., Brown A. (2022). Synthesis, Optimization, and Structure–Activity Relationships of Imidazo[1,2- *a* ]Pyrimidines as Inhibitors of Group 2 Influenza A Viruses. J. Med. Chem..

[29] Argade M.D., Anirudhan V., Bradley S.P., Tomorowicz Ł., Bott R., Sownthirarajan B. (2025). Refinement of Imidazo[2,1-a]Pyrimidines in pursuit of potential drug candidates against group 2 influenza A viruses. Eur. J. Med. Chem..

[30] Du R., Cheng H., Cui Q., Peet N.P., Gaisina I.N., Rong L. (2021). Identification of a novel inhibitor targeting influenza A virus group 2 hemagglutinins. Antivir. Res.

[31] Antanasijevic A., Haferman N.J., Shimon A., Tundup S., Anirudhan V., Rong L. (2025). Inhibition of influenza entry by organosilicon compounds. J. Med. Virol..

[32] Das S., Singh S., Chawla V., Chawla P.A., Bhatia R. (2024). Surface plasmon resonance as a fascinating approach in target-based drug discovery and development. Trac. Trends Anal. Chem..

[33] Copeland R.A. (2016). The drug–target residence time model: a 10-Year retrospective. Nat. Rev. Drug Discov..

[34] Xu R., Wilson I.A. (2011). Structural characterization of an early fusion intermediate of influenza virus hemagglutinin. J. Virol..

[35] Zhou Y., Wu C., Zhao L., Huang N. (2014). Exploring the early stages of the pH-Induced conformational change of influenza hemagglutinin: ph sensing in influenza hemagglutinin. Proteins Struct. Funct. Bioinforma..

[36] Antanasijevic A., Durst M.A., Lavie A., Caffrey M. (2020). Identification of a pH sensor in influenza hemagglutinin using X-Ray crystallography. J. Struct. Biol..

[37] Xu Y., Anirudhan V., Gaisina I.N., Du H., Alqarni S., Moore T.W. (2025). Mechanistic insights into the small-molecule inhibition of influenza A virus entry. Proc. Natl. Acad. Sci..

[38] Caffrey M., Lavie A. (2021). pH-Dependent mechanisms of influenza infection mediated by hemagglutinin. Front. Mol. Biosci..

[39] Laskowski R.A., Swindells M.B. (2011). LigPlot+: multiple ligand–protein interaction diagrams for drug discovery. J. Chem. Inf. Model.

[40] Grimsley G.R., Scholtz J.M., Pace C.N. (2009). A summary of the measured p *K* values of the ionizable groups in folded proteins. Protein Sci..

[41] El-Shesheny R., Feeroz M.M., Krauss S., Vogel P., McKenzie P., Webby R.J. (2018). Replication and pathogenic potential of influenza A virus subtypes H3, H7, and H15 from free-range ducks in Bangladesh in mammals. Emerg. Microbes Infect..

[42] Vauquelin G. (2016). Effects of target binding kinetics on *in vivo* drug efficacy: k_off_, k_on_ and rebinding. Br. J. Pharmacol..

[43] Markgren P.-O., Schaal W., Hämäläinen M., Karlén A., Hallberg A., Samuelsson B. (2002). Relationships between structure and interaction kinetics for HIV-1 protease inhibitors. J. Med. Chem..

[44] Vauquelin G. (2018). Link between a high *k*_on_ for drug binding and a fast clinical action: to be or not to be?. Med. Chem. Comm..

[45] Copeland R.A., Pompliano D.L., Meek T.D. (2006). Drug–target residence time and its implications for lead optimization. Nat. Rev. Drug Discov..

[46] Tummino P.J., Copeland R.A. (2008). Residence time of receptor−Ligand complexes and its effect on biological function. Biochemistry.

[47] Núñez S., Venhorst J., Kruse C.G. (2012). Target–drug interactions: first principles and their application to drug discovery. Drug Discov. Today.

[48] Singanayagam A., Zambon M., Barclay W.S. (2019). Influenza virus with increased pH of hemagglutinin activation has improved replication in cell culture but at the cost of infectivity in human airway epithelium. J. Virol..

[49] Basu A., Antanasijevic A., Wang M., Li B., Mills D.M., Ames J.A. (2014). New small molecule entry inhibitors targeting hemagglutinin-mediated influenza A virus fusion. J. Virol..

[50] White K.M., De Jesus P., Chen Z., Abreu P., Barile E., Mak P.A. (2015). A potent anti-influenza compound blocks fusion through stabilization of the prefusion conformation of the hemagglutinin protein. ACS Infect. Dis..

[51] Yang X., Sun H., Zhang Z., Ou W., Xu F., Luo L. (2023). Antiviral effect of ginsenosides Rk1 against influenza a virus infection by targeting the hemagglutinin 1-Mediated virus attachment. Int. J. Mol. Sci..

[52] Harrison S.C. (2008). Viral membrane fusion. Nat. Struct. Mol. Biol..

[53] Lin X., Noel J.K., Wang Q., Ma J., Onuchic J.N. (2016). Lowered pH leads to fusion peptide release and a highly dynamic intermediate of influenza hemagglutinin. J. Phys. Chem. B.

[54] Casalino L., Seitz C., Lederhofer J., Tsybovsky Y., Wilson I.A., Kanekiyo M. (2022). Breathing and tilting: mesoscale simulations illuminate influenza glycoprotein vulnerabilities. ACS Cent. Sci..

[55] Salata C., Calistri A., Alvisi G., Celestino M., Parolin C., Palù G. (2019). Ebola virus entry: from molecular characterization to drug discovery. Viruses.

[56] Jackson C.B., Farzan M., Chen B., Choe H. (2022). Mechanisms of SARS-CoV-2 entry into cells. Nat. Rev. Mol. Cell Biol..

[57] Arrowsmith J. (2011). Phase II failures: 2008–2010. Nat. Rev. Drug Discov..

[58] Waring M.J., Arrowsmith J., Leach A.R., Leeson P.D., Mandrell S., Owen R.M. (2015). An analysis of the attrition of drug candidates from four major pharmaceutical companies. Nat. Rev. Drug Discov..

[59] IJzerman A.P., Guo D. (2019). Drug–target association kinetics in drug discovery. Trends Biochem. Sci..

[60] Olaru A., Bala C., Jaffrezic-Renault N., Aboul-Enein H.Y. (2015). Surface plasmon Resonance (SPR) biosensors in pharmaceutical analysis. Crit. Rev. Anal. Chem..

