# Supplementary material for: Effect of pH on small-molecule inhibitor binding to influenza virus hemagglutinin
Source: J Biol Chem. 2026 Jan 10;302(3):111150. doi: 10.1016/j.jbc.2026.111150 (PMC12887400; doi:10.1016/j.jbc.2026.111150)
Supplement: Supporting information [file mmc1.docx]

**Supporting Information**

**Effect of pH on small-molecule inhibitor binding to influenza virus hemagglutinin**

Varada Anirudhan^1*^, Irina Gaisina^2,3^, Amir Shimon^4^, Hyun Lee^2,5^, Saad Alqarni^6^, Balaji Manicassamy^7^, Terry W. Moore^8^, Kai Xu^9,10^, Michael Caffrey^4*^, Lijun Rong^1,3*^

^1^Department of Microbiology and Immunology, University of Illinois Chicago, Chicago, IL 60612, USA

^2^Department of Pharmaceutical Sciences and UICentre, University of Illinois Chicago, Chicago, IL 60612, USA

^3^Chicago BioSolutions Inc., Chicago, IL 60612, USA

^4^Department of Biochemistry and Molecular Genetics, College of Medicine, University of Illinois Chicago, Chicago, IL 60607, USA

^5^Biophysics Core at Research Resource Center, University of Illinois Chicago, Chicago, IL

60607, USA

^6^Department of Pharmaceutical Chemistry, College of Pharmacy, University of Ha’il, Hail 55473, Saudi Arabia

^7^Department of Microbiology and Immunology, University of Iowa, Iowa City, IA 52242, United States

^8^UI Cancer Center, University of Illinois Chicago, Chicago, IL 60612, USA

^9^Department of Veterinary Biosciences, The Ohio State University, Columbus, OH 43210, USA

^10^Department of Microbial Infection and Immunity, The Ohio State University, Columbus, OH 43210, USA

^*^Corresponding Authors: vaniru2@uic.edu, caffrey@uic.edu, lijun@uic.edu


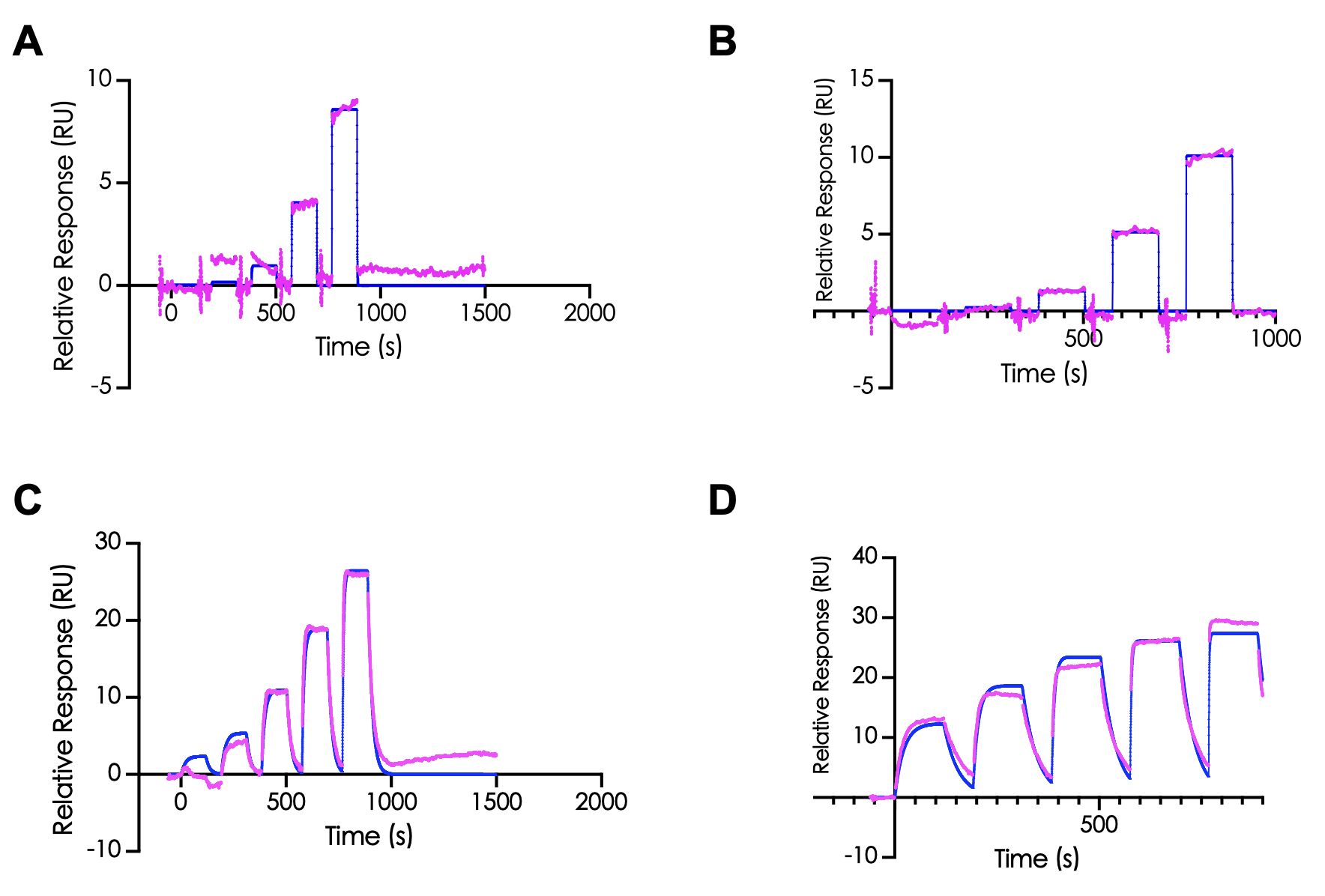


**Figure S1:** Representative sensorgrams from SPR runs to evaluate the kinetic parameters of the compounds ING-1636 and SA-67 binding to H3 and H7 HA at pH 7.2: (A) H3 HA-ING-1636 (B) H3 HA-SA-67 at pH 7.2, (C) H7 HA-ING-1636 at pH 7.2, (D) H7 HA-SA-67 at pH 7.2.

**
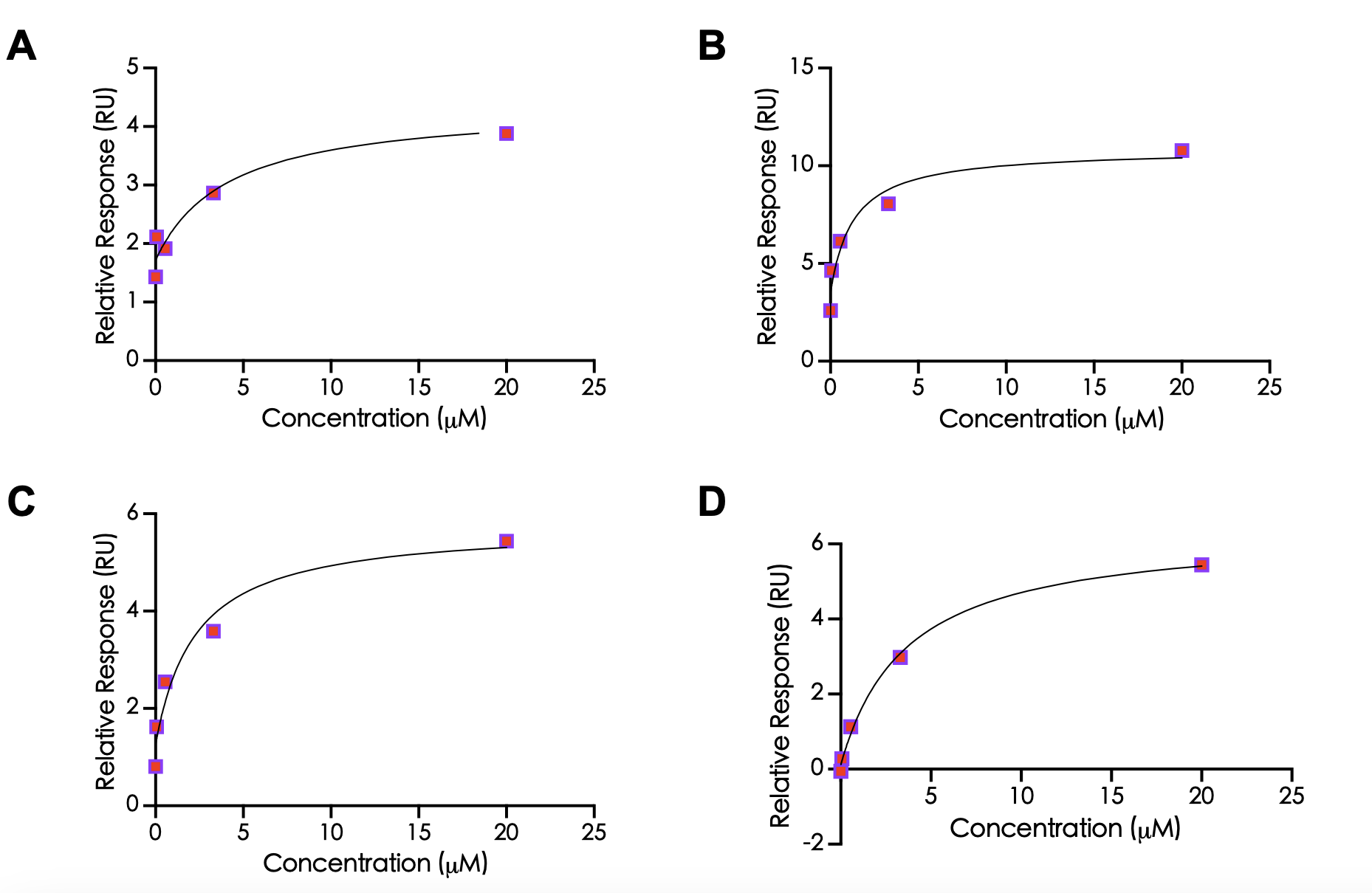
**

**
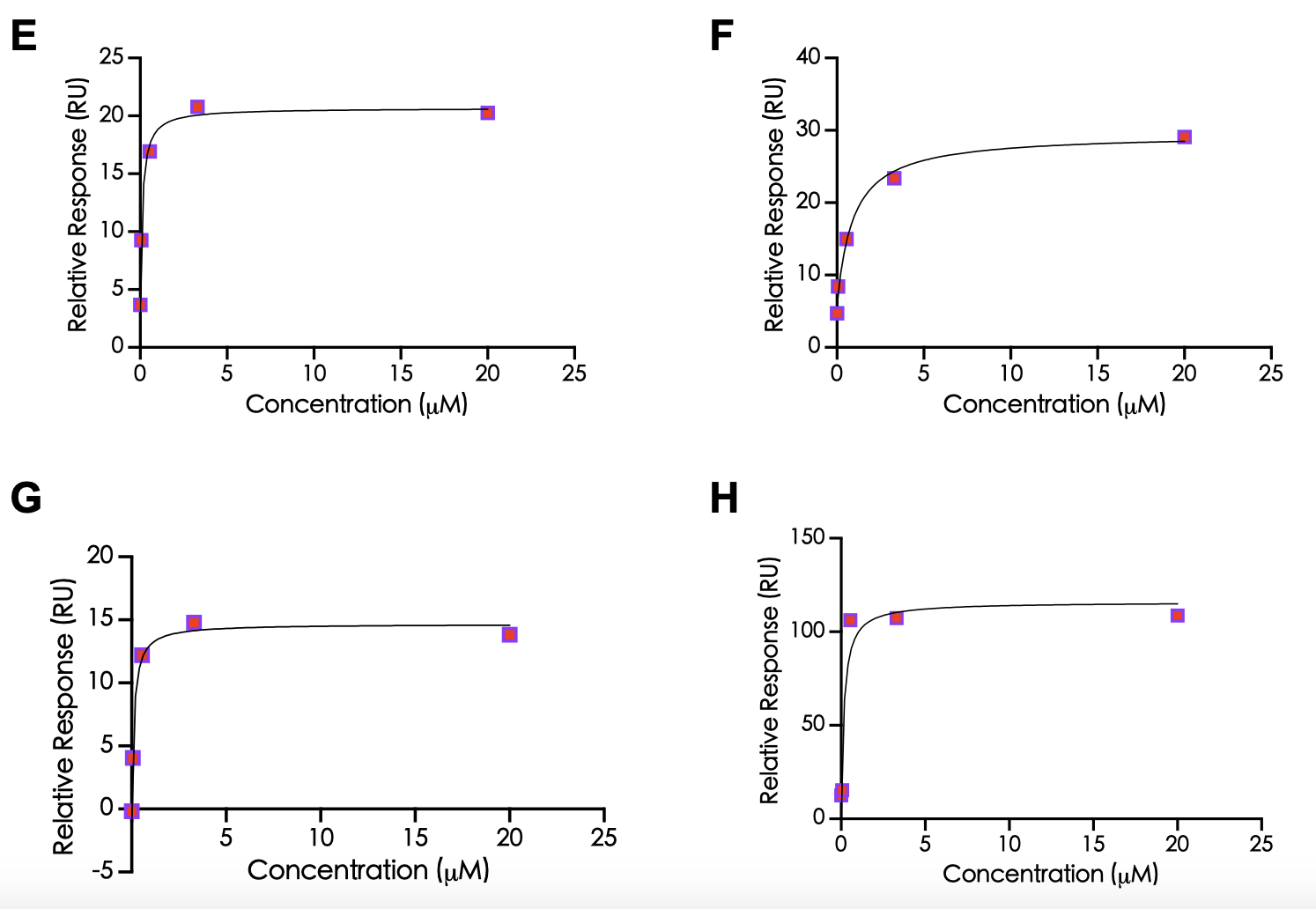
**

**Figure S2:** Representative binding affinity graphs from SPR runs to evaluate pH dependence of K_D_ and residence time of the compounds ING-1636 and SA-67. (A) H3 HA-ING-1636 at pH 6.4, (B) H3 HA-ING-1636 at pH 6.2, (C) H3 HA-SA-67 at pH 6.4, (D) H3 HA-SA-67 at pH 6.2, (E) H7 HA-ING-1636 at pH 6.4, (F) H7 HA-ING-1636 at pH 6.2, (G) H7 HA-SA-67 at pH 6.4, and (H) H7 HA-SA-67 at pH 6.2

**
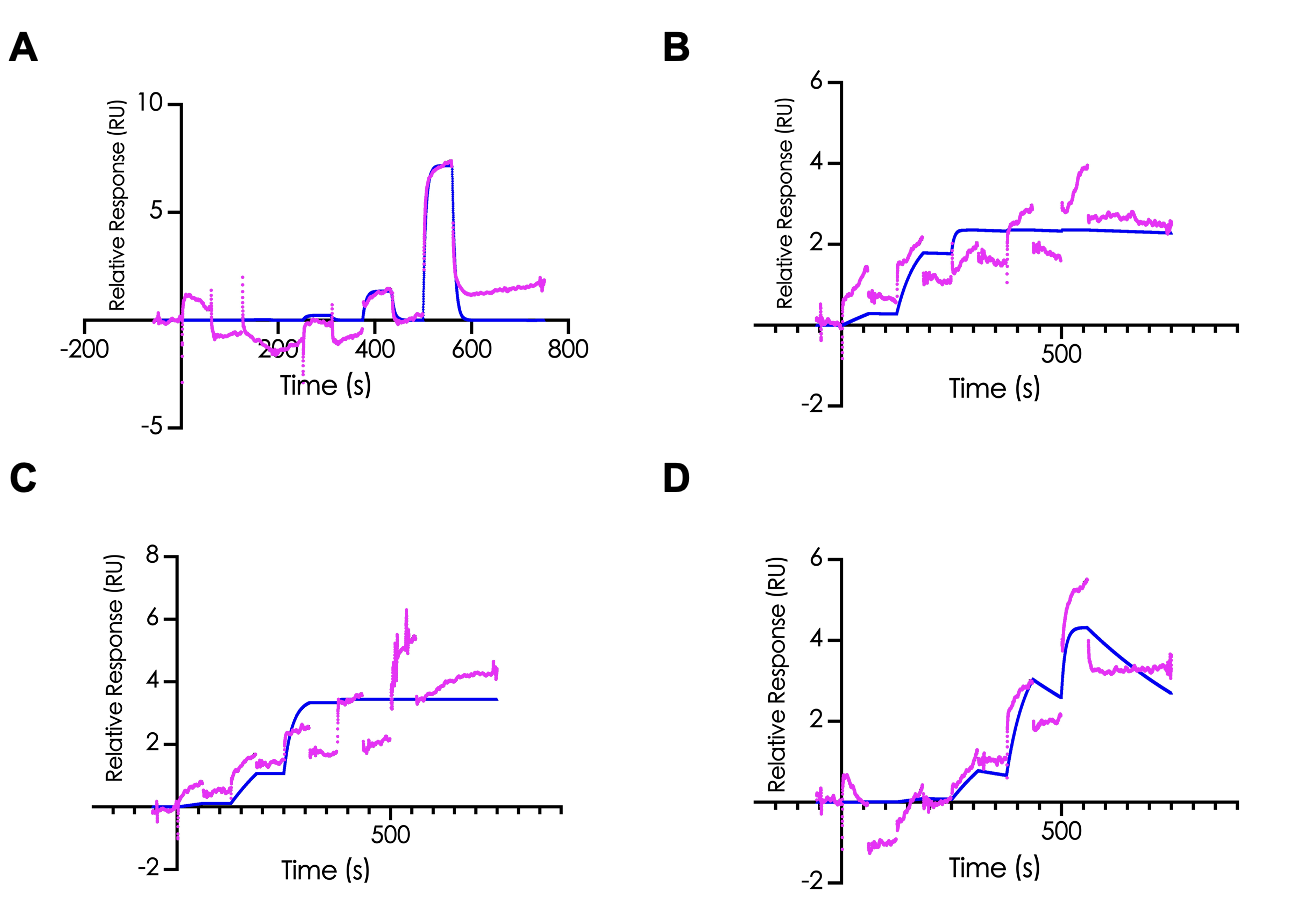
**

**
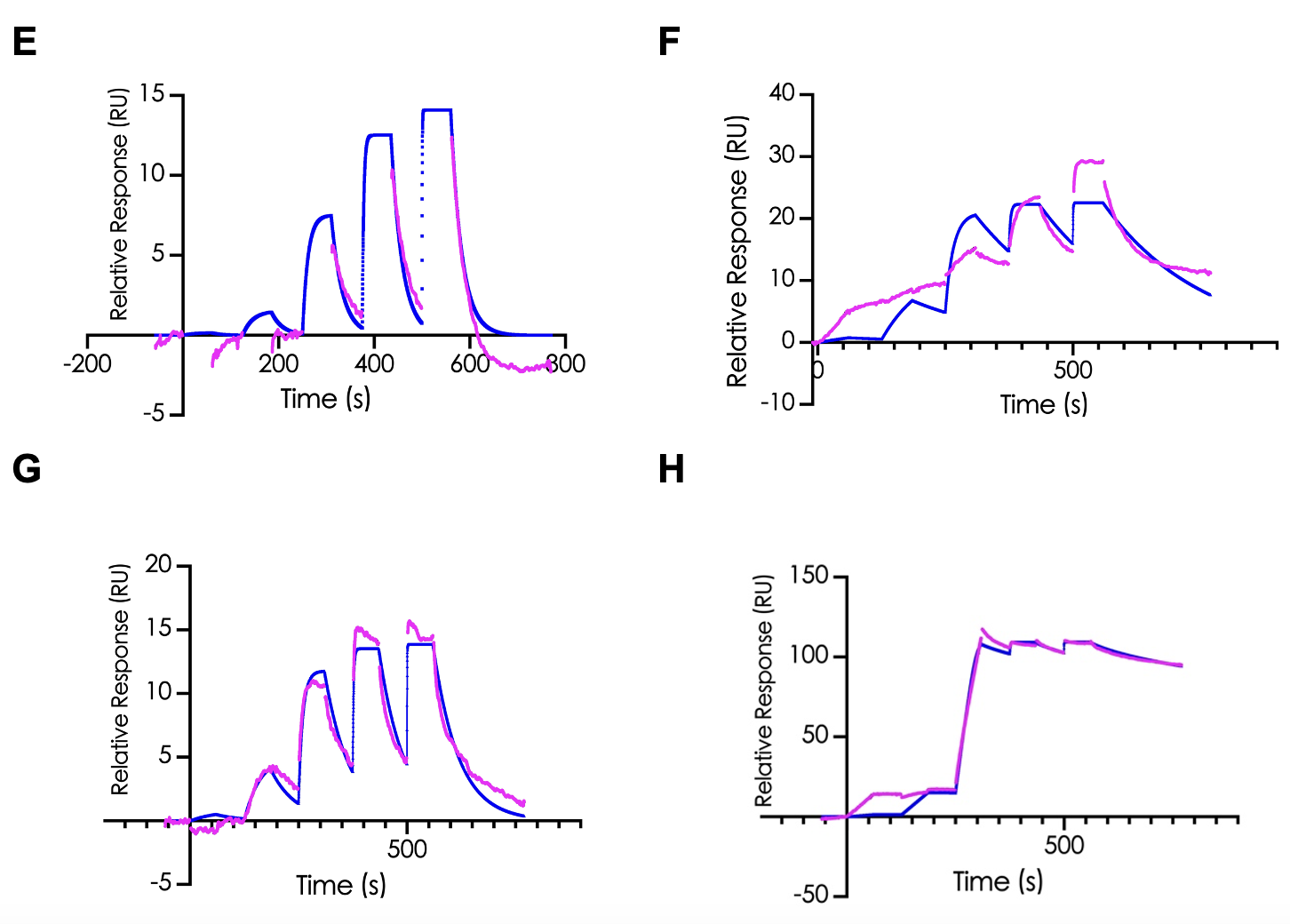
**

**Figure S3:** Representative sensorgrams from SPR runs to evaluate the kinetic parameters of the compounds ING-1636 and SA-67 binding to H3 and H7 HA at pH values 6.4 and 6.2: (A) H3 HA-ING-1636 at pH 6.4, (B) H3 HA-ING-1636 at pH 6.2, (C) H3 HA-SA-67 at pH 6.4, (D) H3 HA-SA-67 at pH 6.2, (E) H7 HA-ING-1636 at pH 6.4, (F) H7 HA-ING-1636 at pH 6.2, (G) H7 HA-SA-67 at pH 6.4, and (H) H7 HA-SA-67 at pH 6.2.
